# Revisiting Polymer–Particle Interaction in PEO Solutions

**DOI:** 10.1021/acs.langmuir.0c02715

**Published:** 2021-03-25

**Authors:** A. Espasa-Valdepeñas, J. F. Vega, V. Cruz, J. Ramos, A. J. Müller, J. Martinez-Salazar

**Affiliations:** † Biophym, Departamento de Física Macromolecular, Instituto de Estructura de la Materia (IEM-CSIC) c/Serrano 113 bis, 28006 Madrid, Spain; ‡ POLYMAT and Department of Polymers and Advanced Materials: Physics, Chemistry and Technology, Faculty of Chemistry, University of the Basque Country UPV/EHU, Paseo Manuel de Lardizabal 3, 20018 Donostia-San Sebastián, Spain; § IKERBASQUE, Basque Foundation for Science, 48009, Bilbao, Spain

## Abstract

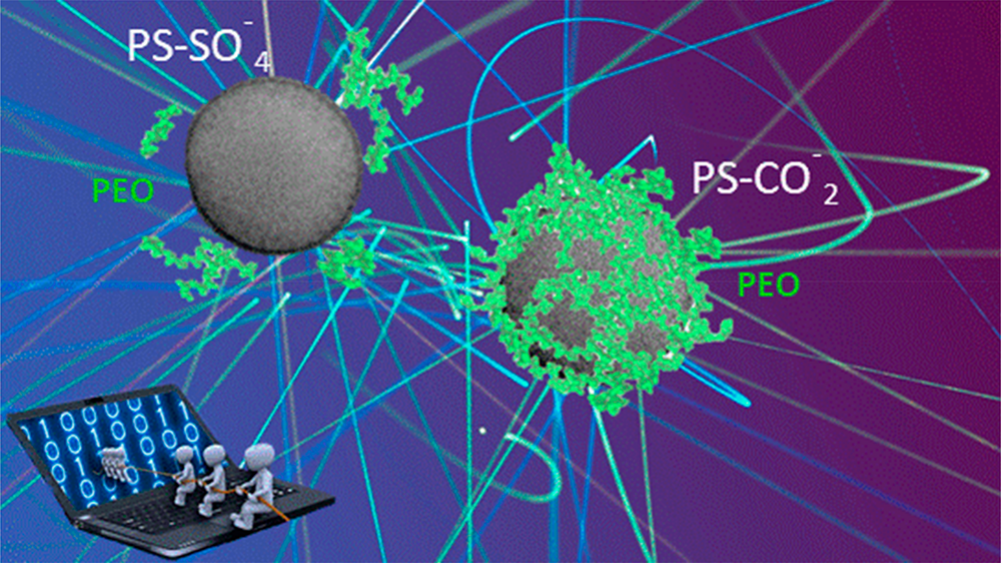

We have measured the electrophoretic mobility and diffusion coefficient of carboxylate-modified and sulfate-modified polystyrene latex particles in poly(ethylene oxide) aqueous solutions. *Carboxylate*-modified polystyrene particles have shown a bound polymeric layer as the surface net charge vanishes even at very low poly(ethylene oxide) concentration. The polymeric layer causes a lower electrophoretic mobility and slower Brownian diffusion than that corresponding to the bare particles. We show that the diffusion is the result of a significantly increased effective particle size 2*r*_*h*__eff_ = 30 nm. This bound layer is not present in sulfate-modified polystyrene latex particles. The interaction between the carboxylate-modified particle surface and the macromolecules has been confirmed by means of atomistic computer simulations. The grafted acrylate copolymers, which come from the preparation procedure of the latex particles, confer more hydrophobic surface ready to interact with the polymer. The simulations suggest that the interaction is modulated not only by the nature of the acrylic acid monomer but also by the length of the grafted copolymer. Our results have important implications for particle selection in microrheology experiments.

## Introduction

The study of the diffusion of biomacromolecules and micro- or nanoparticles in polymeric media (solutions or in the molten state) has not only basic implications, but also a great interest from the point of view of industrial, medical, and biotechnological applications.^1,2^ In this context, the development of microrheological techniques has been a great advance, when applied to systems such as polymeric solutions, polymers in the molten state, gels, and biological systems.^3−6^

In the case of active microrheology, the force exerted on the probe particles is external (magnetic and laser tweezers). The specific case of passive microrheological techniques are based on the monitoring of the Brownian motion of spherical particles, in order to relate this motion with the viscoelastic characteristics of the surrounding medium, by generalizing the well-known Stokes–Einstein relation.^7^ A challenge in passive microrheology using probes is the design of particles with specific surface properties, in order to avoid interactions with the host medium. Polymeric particles in particular, are especially interesting mainly due to their versatility, tunable sizes, physical properties and low toxicity. The interfacial phenomena on the surface with the local environment may already alter the particle transport in complex media such as polymeric or biological fluids. Also, the size of the probe is important, and, in order to extract the correct viscoelastic fingerprint of the medium, it must be larger than the radius of gyration, *r*_g_, of the polymeric chain.^8^ Interestingly, the use of particles with a size lower than the correlation length might give access to the local properties of the system.^9^ Notwithstanding, it should be noted at this respect that the question of the probe size effect in microrheology is still open, but it may be exploited to extract interesting features of complex systems at the local scale.

Within all the broad literature studies concerning microrheological approaches to study complex systems, we have selected those performed in poly(ethylene oxide) (PEO) aqueous solutions, as they have been used as models for the development of passive microrheological techniques based on diffusing wave spectroscopy (DWS) and dynamic light scattering (DLS). It should be noted that the tracer diffusion of micro- and nanosized particles in polymeric solutions has attracted a great deal of attention from the experimental point of view since the 80s.^10−14^ Most of these works are focused on the determination of the diffusion coefficient, *D*, of tracer particles of radius *r* in polymeric solutions, the subsequent application of the Stokes–Einstein relation *D* = *k*_B_T/6πηr to obtain the viscosity of the medium, η, for the subsequent comparison to that measured by mechanical rheology, in most of the cases. The early studies of Phillips et al. in the 80s were performed by determining the diffusion coefficient of *carboxylated* polystyrene latex particles in PEO solutions (among other polymers).^10−12^ In these works, the authors already determined the existence of the “overlapping” and “entangled” regimes as PEO concentration increases, but they also reported the failure of the Stokes–Einstein relationship and the appearance of fast modes in the light scattering spectrum in solutions prepared with the highest molecular weight PEO samples. Remarkably, the authors were able to interpret the measured diffusion coefficients in terms of non-Stokes–Einstein hydrodynamics and the *adsorption* of the polymeric chains by the optical probes.

In the 90s, Mason and Weitz presented their generalization of the Stokes–Einstein relationship to viscoelastic fluids.^7^ These authors used a generalized Langevin equation to relate the measured mean-squared displacement of the probes, ⟨*r*^2^(*t*)⟩, obtained by light scattering, to the complex modulus of the fluid, G*(ω), in an experimental framework applied to various systems including a specific PEO/water solution and *polystyrene latex* particles. Since the year 2000, various works have been focused in PEO/water solutions in order to check the validity of the microrheology, using both *carboxylate-modified* and *sulfate* polystyrene latex probes of different sizes.^15,16^ In these works, the effect of surface chemistry is studied indirectly, by comparing the values of ⟨*r*^2^(*t*)⟩ obtained for a given PEO solution, usually in the entangled regime, using probes with the same chemistry and different sizes (around 0.3 and 2 μm), and comparing the results with those obtained by mechanical rheology. The agreement between the microrheological and mechanical results was not always exact, and it could be subject of discussion. Zanten et al. studied aqueous PEO solutions using *sulfate* polystyrene particles in a broader range of concentrations, covering the unentangled and entangled regimes.^17^ These authors also determine the absence of particle–polymer interactions and the minimal influence of longitudinal modes in the Brownian motion by using particles of sizes between 0.2 and 2 μm.

It is clear that the main challenge for an efficient use of microrheological techniques lies on the ability to choose suitable particles to probe the rheological response, that is, those minimizing the particle-sample interaction. The use of more than one particle of the same nature and different sizes is also a quite robust approach,^18^ as the mutual fluctuations of both types of probe particles provide an accurate method to determine not only the viscoelastic properties of the suspending medium, but also to study the boundary conditions of the particle/matrix system and inertial effects.^19−21^ Another appropriate property to detect the interaction between particles and surrounding macromolecules is the electrophoretic mobility. If the surface properties of the particles are already altered by adsorbed macromolecules to the surface, this property will substantially change.^22^ In this work, we have selected *sulfate* and *carboxylate-modified* polystyrene latex particles in order to study their suitability to microrheological studies in PEO/water solutions. We have combined both experiments (electrophoretic mobility and light scattering) and computer atomistic simulations of the systems in order to determine the nature of the particles/polymer interactions.

## Materials and Methods

### Materials and Characterization

The poly(ethylene oxide) (PEO) sample of M_w_ ∼ 300 kg·mol^–1^ was obtained from Sigma-Aldrich. Dust-free PEO aqueous solutions (*c* < 1.5 mg·mL^–1^) were prepared with deionized water that had been filtered through 0.02 μm syringe filters (Whatman-Anotop 25). The weight-average molecular weight, *M*_w_, and the second virial coefficient, *B*_22_, of the PEO sample were determined by static light scattering (SLS) using the Zetasizer Nano ZS apparatus. The measurements for different sample concentrations in the dilute regime were obtained at *T* = 298 K. In addition to the SLS measurements, dynamic light scattering (DLS) experiments and cumulant analysis have been performed at each concentration using the same apparatus, in order to estimate the diffusion coefficient, *D*, and the hydrodynamic size, *r*_h_, of the polymer coils. In both types of experiments polystyrene cuvettes (Malvern Instruments DTS0012) have been used.

Polystyrene (PS) latex spheres bearing sulfate surface groups (PS-SO_4_^–^) of nominal radius 30 and 100 nm and carboxyl surface groups (PS-CO_2_^–^) of nominal radius of 150 nm were utilized as optical probes (Duke Scientific, Palo Alto, CA). The optical probes at 0.1–0.01% v/v were dispersed in water and in solutions with variable concentration of PEO (*c* ≪ 1.0 mg·mL^–1^) for DLS and electrophoretic mobility (EM) measurements at *T* = 298 K. EM was measured also in the Zetasizer Nano ZS apparatus, which uses phase analysis light scattering (PALS). The universal dip cell with palladium electrodes (Malvern Instruments ZEN1002) were used to perform the measurements. Charged particles are attracted to the oppositely charged electrode, and their velocity was measured and expressed per unit field strength as the EM, μ_e_. The measured conductivity of the solutions remains nearly constant and low, around 0.053 ± 0.017 mS/cm.

The molecular, hydrodynamic, and electrostatic properties obtained at *T* = 298 K for the nanoparticles and the PEO sample under study are listed in Table 1. The specific details of the experimental procedures and materials characterization can be found in the Supporting Information (SI).

**Table 1:** Hydrodynamic and Electrostatic Properties of the Systems under Study at *T* = 298 K

sample	*D* μm^2^·s^–1^ (z-average)	*r*_h_ (*r*_g_) nm (z-average)	*Q*	μ_e_ μm·cm·V^–1^s^–1^
PEO300	8.55	28.7 (41.6)^a^	0.37	–0.52 ± 0.02
PS-SO_4_^–^	7.00	35.0	0.03	–3.2 ± 0.2
PS-SO_4_^–^	2.38	103.0	0.03	–3.8 ± 0.2
PS-CO_2_^–^	1.66	148.0	0.03	–3.6 ± 0.1

a The value between the parentheses corresponds to *r*_g_ for PEO estimated from *r*_g_ = 1.45*r*_h_. Experiments for several polymers in good solvents report *r*_g_ = 1.45*r*_h_.^23^ Analytical theories predict *r*_g_ = 1.59*r*_h_.^24^

### Computer Simulations

#### Simulated Systems

The atomistic structure of the surface of the nanoparticles has been constructed by taking into account the specific features of the real systems (see point 3 of the SI). The large size of the nanoparticle (35–148 nm of radius) and the high molecular weight of the polymer (284.1 kg·mol^–1^) make impractical the atomistic simulations of these systems. For this reason, the atomistic models have been built on a small-scale model able to capture the interactions between the surface of the nanoparticle and the polymer solution. Thus, a central bilayer model representing a small fraction of the nanoparticle surface was built resulting in two interacting sides each mimicking the nanoparticle surface.

In order to assess the effect of the different surfactant/polymer models on the bilayer-polymer interactions, the following simulation systems were built (see SI Scheme S.1): (1) A slab containing 64 polystyrene (PS) oligomers composed of 10 monomeric units, each capped at both ends with SO_4_^–^ groups, was built as an 8 × 8 layer at the center of a simulation box. The system is intended to mimic a surfactant free sulfonated nanoparticle surface (PS-SDS-free). (2) Abilayer of SDS molecules where either 4 (PS-4-SDS) or 9 (PS-9-SDS) PS oligomers are inserted at regular positions mimicking a surfactant saturated nanoparticle surface. The SDS molecules with atoms nearer than 1.5 Å from any PS chain atom were removed from the simulation box. As each oligomer has a SO_4_^–^ group in each terminal monomer, the number of SO_4_^–^ groups per leaflet is 4 and 9, respectively. These systems are intended to mimic the real nanoparticle surface with surfactant as described in the Supporting Information file (see SI Section S.3). (3) In order to model the grafted copolymers present in the *carboxylate*-modified nanoparticles, an H atom of the outer styrene monomers is substituted by a small acrylate copolymer of variable number and monomer composition. The selected functionalities are acrylic acid (MAA), methyl methacrylate (MMA) and n-propyl methacrylate (PMA). Different copolymers have been built to change the ratio monomer surface/charge on the bilayer surface. In fact, the copolymers are grafted on the PS oligomer on the bilayer model, acting as “hairs” on the surfaces. For example, the system O1 is built using MAA, short monomer with negative charge, in which the ratio monomer surface/charge remains low. On the other hand, the O4 models are characterized by a high monomer surface/charge ratio. These systems are intended to mimic the real functionalized nanoparticle surface (PS-CO_2_^–^ system, see SI Section S.3). Na^+^ ions were selected in accordance with the materials used in the experimental part. Although it is already known that other ions, as K^+^, can make a difference,^25^ it may be expected that the hydrophobic driven interaction between nanoparticle and polymer would be similar whether K^+^ ions were considered instead. Table 2 gives the details about the configuration of each system.

**Table 2:** Composition of Each Simulated System^a^

	name	number of PS molecules	number of SDS molecules	CO_2_^–^ grafted oligomer composition on each PS oligomer side
surfactant	**SDS**	0	288	
PS-SO_4_^–^	**PS-SDS free**	64	0	
	**PS-4-SDS**	4	254	
	**PS-9-SDS**	9	125	
PS-CO_2_^–^	**O1–4-SDS**	4	248	3 MAA
	**O1–9-SDS**	9	198	3 MAA
	**O2–4-SDS**	4	248	1 MAA, 2 MMA
	**O2–9-SDS**	9	198	1 MAA, 2 MMA
	**O3–4-SDS**	4	252	1 MAA, 2 MMA, 3 PMA
	**O3–9-SDS**	9	207	1 MAA, 2 MMA, 3 PMA
	**O4–4-SDS**	4	240	2 MAA, 4 MMA, 6 PMA
	**O4–9-SDS**	9	180	2 MAA, 4 MMA, 6 PMA

a CO_2_^–^ based monomers: acrylic acid (MAA), methyl methacrylate (MMA) and n-propyl methacrylate (PMA).

#### Force Field and Simulation Protocol

The well-established OPLS-AA and LOPLS-AA force fields have been used for all the simulations.^26−28^ In particular, improved parameters for the SDS molecules were taken from refs (26−28). The molecular dynamics simulations were performed taking into account the following common options: NPT (*P* = 1 atm and *T* = 300 K). V-rescale thermostat was used in the simulations,^29^ this is an extension to Berendsen coupling by adding a stochastic term that ensures a proper canonical ensemble.

Coulomb potential was evaluated using Particle Mesh Ewald (PME) with a Fourier grid spacing of 0.16. A cutoff of 1 nm was used for coulomb and van der Waals interactions.^30,31^

For each system described in the previous section, the following computational steps were applied:

1. Minimization of the whole system to relief bad contacts or forced molecular topology. The L-BFGS minimization procedure was used with a convergence criterion of maximum force below 10^3^ kJ·mol^–1^·nm^–1^.
2. 1 ns molecular dynamics with position restraint on all solute atoms. The interaction energy between water molecules and solute takes around 200 ps to relax.
3. 20 ns of NPT simulation in the same conditions. The density of the bilayer model stabilized within few nanoseconds of the simulation. The surface area per SDS molecule also converged to a plateau value around the same time. The different interaction energy components stabilize within the same amount of time.
4. The final structure of the previous step is selected for further processing. Solvent molecules and counterions are deleted. Ten PEO oligomers containing 10 monomers each are randomly inserted in the empty box space above and below the slab. The resulting system is resolvated and counterions added again to neutralize the system.
5. Minimization and position restraint dynamics are performed as described in points 2 and 3 of the protocol.
6. Finally, production run is performed. Two replicas of 400 ns NPT molecular dynamics simulation for each system are performed in the conditions above-described.

## Results and Discussion

### Electrophoretic Mobility and Dynamic Light Scattering

**Figure 1:**
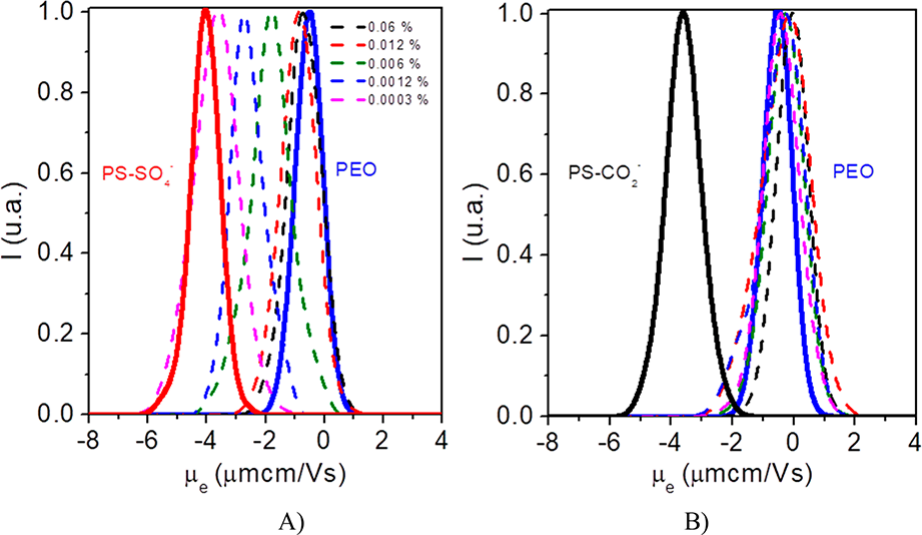
Electrophoretic mobility distribution of bare PEO and PS particles (solid lines) and PEO/PS systems for different PEO300 concentrations (dashed lines) at *T* = 298 K. (A) PEO/PS-SO_4_^–^ (100 nm) and (B) PEO/PS-CO_2_^–^(150 nm).

A full characterization of the PEO sample and the optical probes used in this work has been performed. The specific details can be found in the SI. If an interaction exists between the PEO molecules and the probe particles, the surface net charge of the later should change depending on the degree of particle coverage and on the polymer nature. Additionally, the experiments should be performed at suitable concentrations of both tracer particles and polymer. This means that the polymer concentration should be low enough for the nature of the particle surface not to be screened by the polymer. For this reason, we have performed measurements at a given particle concentration (*c*_p_ = 0.1% w/v) and variable polymer concentration in the dilute regime (*c* ≪ 0.5 mg·mL^–1^). The results obtained for μ_e_ in both PEO/PS-SO_4_^–^ (100 nm) and PEO/PS-CO_2_^–^ (150 nm) systems in the whole PEO concentration range explored are shown in Figure 1. It is observed that in the case of PS-SO_4_^–^ nanoparticles a strong decrease of μ_e_ toward the corresponding value obtained for the bare PS-SO_4_^–^ particles is observed as the PEO concentration decreases, which reflects the lack of interaction between the PEO macromolecules and the sulfate functionalized particles (Figure 1A). In this case the PEO chains screen the probe particles surface charge for polymer concentrations higher than 0.1 mg·mL^–1^, for the selected particle concentration of 0.1% (w/v). Below this threshold an abrupt change of μ_e_ value takes place, and reaches the measured value of the bare PS-SO_4_^–^ for PEO concentrations well below 0.01 mg·mL^–1^. On the contrary, in the case of PS-CO_2_^–^ the values of μ_e_ remains around the corresponding value of PEO in the entire polymer concentration range explored, a result that clearly indicates that the polymer macromolecules are firmly anchored to the particle surface (Figure 1B).

The value of μ_e_ obtained for PEO/PS-CO_2_^–^ systems, even at very low concentration of PEO (*c* ∼ 3.0 × 10^–3^g·mL^–1^), is in agreement with those reported in covalently PEGylated nanoparticles of different nature.^32−34^ It should be recalled here that given the μ_e_ and *r*_h_ values of the bare nanoparticles, the charge surface density is almost the same for both PS-CO_2_^–^ and PS-SO_4_^–^ systems. Also, the hydrodynamic size of both particles is not very different, so in the absence of interactions between PEO chains and particles, the critical concentration to cancel the screening effect of the polymer over the surface charge of the particles should be also similar.

**Figure 2:**
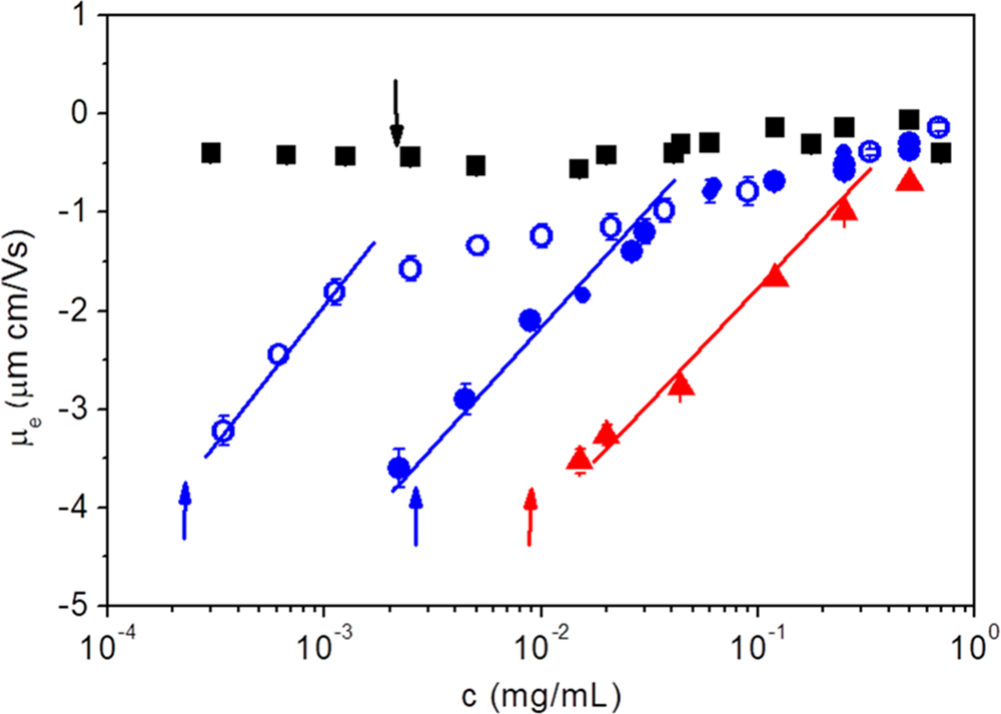
Electrophoretic mobility of the optical probes for different PEO concentrations: (black square) PEO/PS-CO_2_^–^ (150 nm), (closed and open blue circle) PEO/PS-SO_4_^–^ (100 nm), (red triangle) PEO/PS-SO_4_^–^ (30 nm). Close symbols correspond to series with particle concentration *c*_p_ = 0.001 mg·mL^−1^. Open symbols correspond to solutions with particle concentration *c*_p_ = 0.0001 mg·mL^−1^. The arrows indicate the value of *c*_m_ for each system.

This critical concentration should depend in principle on the total surface of the particles, *S*_T_, and then on their size, *r*_p_, and concentration, *c*_p_, but also on the size of polymeric chains, given by *r*_g_. On one hand, we may assume that the necessary number of molecules per volume unit to shield the surface is given by *N*_m_ = *c*_m_*N*_a_/*M*_w_. On the other hand, the total particle surface per unit volume can be obtained by *S*_T_ = *N*_p_·*S*_p_, being *N*_p_ the number of particles per volume unit *N*_p_ = 3*c*_p_/4πr_p_^3^ρ_p_ and *S*_p_ the surface of a particle *S*_p_ = 4π*r*_p_^2^. The ratio between *S*_T_ and *N*_m_ around the critical concentration should be of the order of π*r*_g_^*2*^. At a given particle fractional volume in the solution, *c*_p_/ρ_p_, the critical concentration, *c*_m_, scales as *M*_w_/*r*_p·_*r*_*g*_^2^. This calculation, considering the experimental conditions used here (*c*_p_ = 0.001 mg·mL^–1^) and the particle and PEO hydrodynamic and molecular properties gives rise to a value of *c*_m_ = 2.5 × 10^–3^ mg·mL^–1^ for PEO300 which is satisfactorily close to that obtained from electrophoretic mobility experiments, as it can be observed in Figure 2.

It should be noted that decreasing the particle concentration to *c*_p_ = 0.0001 mg·mL^–1^ leads to a value of *c*_m_ = 2.4 × 10^–4^ mg·mL^–1^ for PEO, a fact also proved experimentally in Figure 2. Additional experiments have been performed by using PS-SO_4_^–^ particles (*c*_p_ = 0.001 mg·mL^–1^) with a hydrodynamic radius of *r*_p_ = 35 nm (see SI archive). The proposed scaling above gives rise in this case to a value of *c*_m_ = 8.3 × 10^–3^ mg·mL^–1^, which is also close to the experimental results shown in Figure 2. These results have a crucial practical importance, as to determine the possibility of interaction between a particular particle/polymer pair from electrophoretic mobility experiments, the specific polymer concentration to avoid macromolecular shielding onto particle surface should be carefully chosen, depending mainly on particle size and concentration.

**Figure 3:**
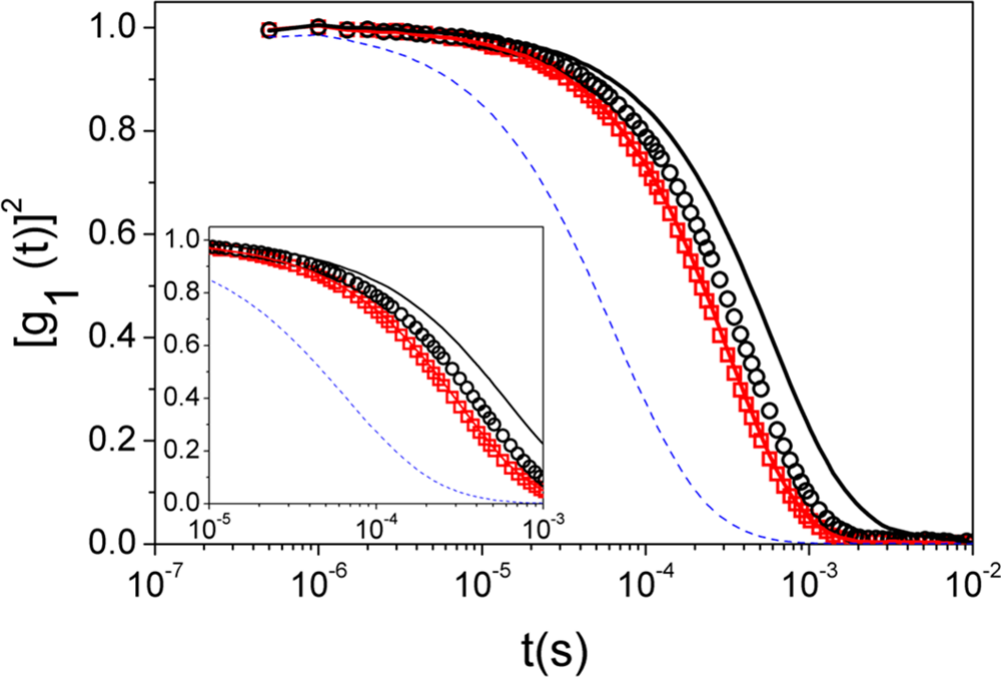
Squared electric field time autocorrelation function, [*g*_1_(*t*)]^2^, of the systems at *T* = 298 K versus time: (dotted line) PEO, (≤, red) PS-SO_4_^–^ (100 nm), (○, black) PS-CO_2_^–^(150 nm), (dashed line, red) PEO/PS-SO_4_^–^, and (solid line, black) PEO/PS-CO_2_^–^. The inset shows a detail of the results in a reduced abscissa range.

PEO is a flexible linear polymer, and if the macromolecules are physically bound to the particle surface, they can dramatically influence the Brownian motion of the PS-CO_2_^–^ particles by introducing additional frictional drag and thus reducing particle diffusivity. Then we have further explored the hydrodynamic properties of the systems in the dilute concentration region, in order to get an additional probe about the polymer/particle interaction. In Figure 3, we can observe the autocorrelation function obtained from DLS experiments in particle–polymer systems for a concentration of PEO of *c* = 2 × 10^–3^ mg·mL^–1^.

It can be clearly observed that the autocorrelation function obtained for the PEO/PS-SO_4_^–^ system is virtually the same as that observed for the bare PS-SO_4_^–^ probe particles (inset in Figure 3). This result is expected if no interaction exists between PEO and PS-SO_4_^–^ particles, assuming that the viscosity of the solution is not greatly affected by such a low concentration of PEO300. On the contrary, the autocorrelation function of the PEO3/PS-CO_2_^–^ system clearly shifts toward higher lag times, a result that indicates a measurable decrease of the diffusion coefficient of the probe particles in identical conditions. The evaluation of the autocorrelation function by means of the cumulant analysis gives a value for the diffusion coefficient *D* = 0.150 μm^2^·s^–1^ and an effective hydrodynamic radius *r*_heff_ = 163.0 nm for the PS-CO_2_^–^ systems. This result is consistent with the presence of a bound PEO layer on the probe surface with a thickness of around 15 nm. It is worth to note that this increase is one-half the hydrodynamic size of PEO chains (see Table 1 and SIe).

### Computer Simulations

Two descriptors are used along this work to characterize the interaction between the bilayer model and the PEO polymer solution. The first one is the so-called “Parking Area” (PA), which represents the average surface area per charge present on that surface:
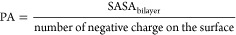
1This descriptor can be stated as a measure of the charge density on the surface. The solvent accessible surface area (SASA) is used in this work to estimate the bilayer and polymer surface areas. The second one is the intermolecular contact area (IA) between the bilayer model and the polymer. This value is calculated as the inaccessible area to solvent owing to the contact between nanoparticle and PEO surfaces. The following equation can be used to calculate the interaction area based on Solvent Accessible Surface Area (SASA) estimations:
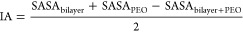
2SASA_bilayer_, SASA_PEO_, and SASA_bilayer+PEO_ are the SASAs calculated for the one leaflet of the bilayer model, the PEO oligomers and the bilayer+PEO system, respectively.

SASA values are needed to calculate both the PA and IA. In this work, SASA is calculated using the method described by Eisenhaber et al., taking into account the approximate water radius (0.14 nm) for the solvent probe.^35^

**Figure 4:**
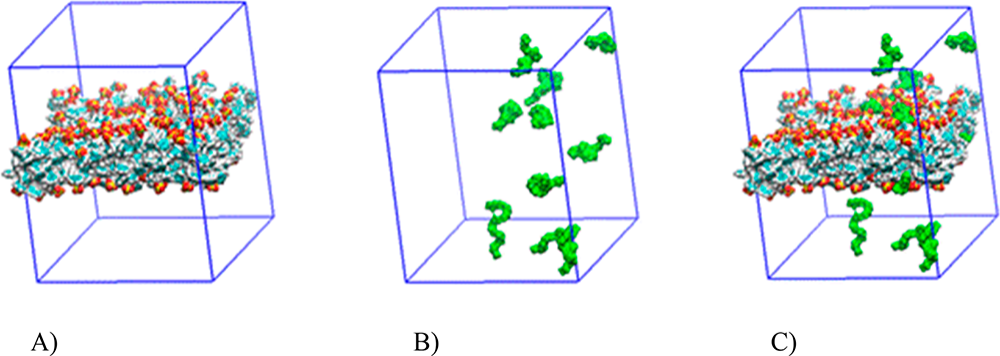
SASA renderings corresponding to a frame of the nonfunctionalized nanoparticle system. (A) nanoparticle (SASA_NP_) (color coded by atom name); (B) PEO oligomers (SASA_PEO_) (green color); and (C) the resulting complex (SASA_NP+PEO_).

**Figure 5:**
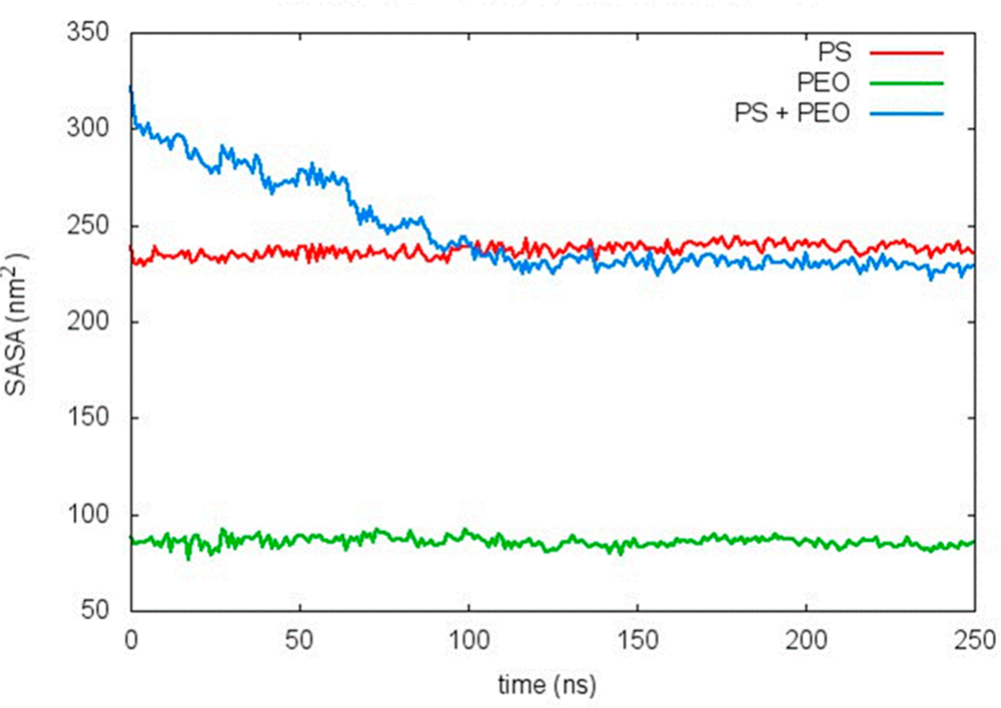
Time evolution along the NPT simulation of SASA corresponding to the nonfunctionalized nanoparticle, PEO oligomers and the NP+PEO complex.

A schematic representation of the IA calculation based on SASA values is shown in Figure 4. Each system is solvated with TIP4P water molecules excluding the hydrocarbon region inside the slab. Counterions are added accordingly to yield neutral systems. For each frame, the SASA is calculated on the slab alone (Figure 4A), on all the PEO oligomers (Figure 4B), and finally on the adsorbed complex and the unadsorbed PEO molecules (Figure 4C). Time evolution of SASA_bilayer_, SASA_PEO_ and SASA_bilayer+PEO_ for PS-SDS-free nanoparticle is illustrated as an example in Figure 5.

Initially, the NP slab and the PEO oligomers are spatially separated so that the SASA_bilayer+PEO_ is approximately the sum of SASA_bilayer_ and SASA_PEO_. As the system evolves, the SASA_bilayer+PEO_ decreases due to the adsorption of PEO molecules on the NP surface. Keep in mind that, this slab corresponds to SO_4_^–^ capped PS oligomers as a representation of the PS-SO_4_^–^ nanoparticle surface. Furthermore, this SASA value can be considered as a measurement of the contact between PEO chains and the nanoparticle. Thus, the separated species show a stable value along the simulation whereas polymer–nanoparticle interactions take around 100 ns to reach a plateau value, after that this value keeps stable. Similar observations were obtained with the other replicas and/or systems considered in this study (not shown).

Two pure bilayer are first simulated, one containing only SDS molecules (SDS) and one composed by PS-SO_4_^–^ oligomers (PS-SDS free). The simulation of SDS is due to the fact that it is the surfactant used in the synthesis of polymer nanoparticle (see SI S3). The SDS slab presents a PA of 0.57 ± 0.02 nm^2^/charge group, slightly larger than the value reported for the estimated area per SDS molecule at adsorption saturation (0.52 nm^2^).^37,38^ Furthermore, the average IA corresponding to the last 100 ns of the PEO-included MD simulation for this system gives a value near 0 (0.2 ± 0.3 nm^2^), that is, there is no interaction between the polymer and the SDS slab. Likely, this is due to the high order observed on the bilayer surface, in which the SO_4_^–^ groups are homogeneously distributed on the surface (low PA value) screening the hydrophobic interactions between SDS and PEO chains.

On the other hand, the PS-SDS free model gives a value of PA around 1.87 ± 0.02 nm^2^/charge group, pointing to a decrease of the charge density on the surface. The atomistic simulation of the interaction between PS-SDS free and the PEO oligomer chains yields an IA of 47 ± 1 nm^2^. This value can be compared with the calculated SASA for the 10 PEO oligomers (84 ± 4 nm^2^). The IA value is more than half the value of the total accessible surf ace of the PEO molecules, being indicative of a strong interaction between the two compounds. This interaction can be visualized in Figure 7A, where most of the PEO chains are in close contact with the hydrophobic areas of the bilayer model. Hence, PEO chains are able to enter through the charged slab to reach the hydrophobic backbone of the PS chains. In principle, this is in contrast to the lack of interaction between the PS-SO_4_^–^ and the PEO polymer observed in the experimental section.

**Figure 6:**
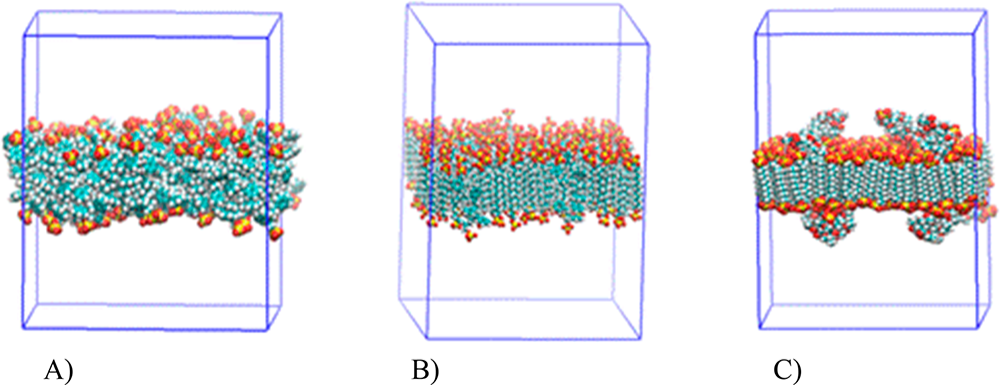
Representation of the simulation box for the NP slabs after 20 ns equilibration. Atoms are represented by VDW spheres colored by atom name. (A) Set of 64 PS oligomers (PS-SDS free); (B) 4 PS oligomers inserted in a SDS bilayer (PS-4-SDS); (C) 4 grafted CO_2_^–^ copolymers on PS chains inserted in a SDS bilayer (O3–4-SDS) (see text and Table 3). Water molecules and counterions are not shown.

As a consequence of the emulsion polymerization process used to synthesize the PS nanoparticles, SDS molecules can remain trapped on the nanoparticle surface with the sulfate moieties exposed to the hydrophilic phase (see SI Section S.3). Therefore, we have simulated the nonfunctionalized PS-SO_4_^–^ systems as slabs containing SDS molecules and PS oligomers. The SDS:PS oligomers ratio considered in the model bilayer systems can be estimated in the following way: On one hand, the nanoparticle surface is around 1.25 × 10^5^ nm^2^ given the nanoparticle sphere diameter of 200 nm. Taking into account the estimated area per SDS molecule at adsorption saturation (0.52 nm^2^), we obtain around 2.4 × 10^5^ SDS molecules on the nanoparticle surface. On the other hand, given the density of polystyrene (ρ = 1.05 g·cm^–3^) the nanoparticle volume (V ∼ 4 × 10^6^ nm^3^), and the PS molecular weight (*M*_w_ ∼ 300 kg·mol^–1^) the number of PS molecules can be calculated with the following formula:
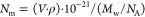
3where *N*_A_ is the Avogadro’s number and *N*_m_ is the number of PS molecules in the nanoparticle. The numerator accounts for the nanoparticle mass whereas the denominator accounts for the PS molecule weight, both expressed in grams. This results around 8000 PS molecules on each nanoparticle, giving a proportion of 30 SDS molecules per PS chain. As shown in Table 2, we have built two sets of systems with a ratio of around 30, close to the above estimation, and 10, approximately. The close packing of SDS surfactant molecules forms a bilayer in which the PS oligomers are embedded with SO_4_^–^ groups exposed at the interface with the hydrophilic medium (Figure 6B).

**Figure 7:**
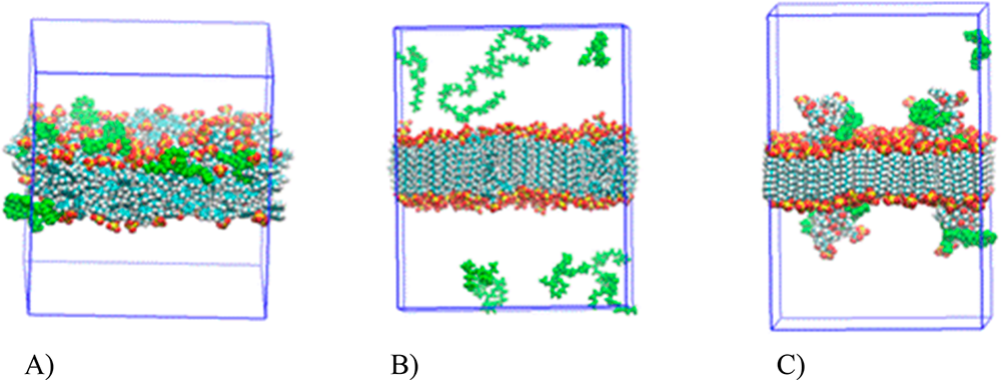
Representation of the simulation box for different NP slabs including its interaction with the PEO oligomers. Final snapshots after 400 ns NPT dynamics. Atoms are represented by VDW spheres colored by atom name. PEO oligomers are also depicted as VDW spheres in green color. (A) Set of 64 PS oligomers. (B) Four PS oligomers inserted in a SDS bilayer. (C) Four grafted CO_2_^–^ copolymers on PS chains inserted in a SDS bilayer (see text). Water molecules and counterions are not shown.

The PS-SO_4_^–^ systems presents a large ordering in the hydrophobic region due to the packing of all-trans SDS hydrocarbon chains. This observation is in agreement with recent experimental findings on SDS bilayers by Nakamura et al.^39^ Those authors identified all trans conformation in the hydrocarbon chains corresponding to a closed packing of SDS molecules, using a combination of infrared external spectra, atomic force microscopy (AFM), and quartz crystal microbalance (QCM) techniques. Thus, for example, a slab containing four PS oligomers inserted in a bilayer of 254 SDS molecules (PS-4-SDS) presents an average PA of 0.61 ± 0.01 nm^2^/charge group, evaluated during the last 10 ns of the slab equilibration simulation in the system without the PEO oligomers (Figure 7B). The averaged IA value gives a value near 0 (0.2 ± 0.3 nm^2^), i.e., there is no interaction between the polymer and the particle slab (Figure 7B). The surface is crowded with enough negative charges to prevent hydrophobic interaction between the surface and the PEO oligomers. By increasing the number of PS chains into the SDS bilayer (PS-9-SDS) both the PA and IA increase up to 0.80 ± 0.01 nm^2^/charge group and 7 ± 2 nm^2^, respectively. Although, the IA is increased respect to the system PS-4-SDS, it remains sufficiently low to consider that there is not interaction between the polymer and the NP model.

Regarding the PS-CO_2_^–^ functionalized latex nanoparticles, several combinations of grafted oligomer compositions and SDS:PS ratios were taken into account (Table 2). The grafted oligomers were inserted in a SDS so that the hydrophilic groups interact with the water phase (Figure 7C). The acrylic acid groups form a kind of multianionic “hairs” spread over the nanoparticle surface. It is expected that the number of charges increases respect to the nonfunctionalized nanoparticle due to the contribution of each charged monomer. However, at the same time, the accessible surface increases too due to the exposition of the copolymer chains to the hydrophilic phase. To take into account this, we have built four different functionalized models (O1–O4). The O1 and O2 models contain charged MAA and MMA monomers. These systems have a low ratio surface area/charge. In these systems, the interaction with the PEO polymer is very similar to the interaction already discussed for the PS-SO_4_^–^ system.

The SDS:PS ratio is around 30 on each bilayer face, which is a proportion comparable with the real nanoparticle ratio area taking into account the nanoparticle surface (1.25 × 10^5^ nm^2^), the number of PS chains per nanoparticle (≈ 10^4^) and the estimated area per SDS molecule at adsorption saturation (0.52 nm^2^). The average IA corresponding to the last 100 ns of the PEO-included MD simulation for this system gives a value near 0 (0.2 ± 0.3 nm^2^), that is, there is no interaction between the polymer and the particle slab (Figure 7B). The surface is crowded with enough negative charges to prevent hydrophobic interaction with the PEO oligomers. Taking into account the results of all our simulations, this PA value seems to be an upper limit for the charge density on the particle surface to avoid interaction with PEO. However, the copolymers used experimentally are a mix of acrylate monomers. To illustrate this, we have built a system containing four PS molecules grafted on each side with a copolymer formed by 1 MAA, 2 MMA, and 3 PMA monomers. The PA for an equilibrated layer of this composition is 0.75 ± 0.01 nm^2^/charge group and the resulting IA with the PEO oligomers is 11 ± 4 nm^2^. In this case the hairy structure conferred by the grafted copolymers presents more hydrophobic surface contributed by the uncharged carboxyl monomers ready to interact with the polymer.

Figure 7C shows the final snapshot of this simulation depicting the specific interaction between the grafted copolymer chains and the PEO oligomers. Our calculations suggest that the higher the number of charged acrylic acid monomers that are included in the grafted copolymer composition, the lower the PA and consequently the lower the interaction with the polymer. This can be illustrated by the O1–4-SDS system composed by four PS oligomers each grafted with three charged MAA monomers on both slab sides and inserted into the SDS bilayer. In this case the PA is 0.60 ± 0.01 nm^2^/charge group and the resulting IA is negligible (0.2 ± 0.3 nm^2^, see Table 3). It can also be observed that the larger the fraction of PS oligomers in the slab composition, the larger the IA with the PEO oligomers. In addition, the length increment of grafted *carboxylated* copolymer contributes positively to the PA and consequently to the interaction with the PEO molecules. It should be noted that we have performed additional simulations with PEO chains containing 100 monomers instead of the 10 monomer PEO system. The results obtained for the SDS and PS-SDS free slabs have been very similar to those reported in Table 3. Therefore, we conclude that the 10 PEO oligomeric system is suitable to perform the study as the equilibration of those molecules is substantially faster than the larger polymer.

**Table 3:** Parking Area and Interaction Area for Each System Described in Table 2

system	name	parking area (Å^2^)	interaction area (nm^2^)
Surfactant	SDS	57 ± 2	0.2 ± 0.3

PS-SO_4_^–^	PS-SDS free	187 ± 2	47 ± 1
	PS-4-SDS	61 ± 1	0.2 ± 0.3
	PS-9-SDS	80 ± 1	7 ± 2

PS-CO_2_^–^	O1–4-SDS	60 ± 1	0.2 ± 0.3
	O1–9-SDS	66 ± 1	5 ± 2
	O2–4-SDS	68 ± 1	5 ± 1
	O2–9-SDS	81 ± 1	6 ± 3
	O3–4-SDS	75 ± 1	11 ± 4
	O3–9-SDS	95 ± 2	19 ± 2
	O4–4-SDS	93 ± 1	36 ± 2
	O4–9-SDS	129 ± 2	41 ± 3

## Conclusions

Using light scattering, we measure the electrophoretic mobility and diffusion coefficient of *carboxylate*-modified and *sulfate* latex particles in PEO solutions. The results show a bound PEO layer on the *carboxylate-*modified polystyrene particles, as the surface net charge vanishes even at very low PEO concentration. In addition, this layer causes a slower diffusion than that corresponding to the bare particles. We show that the diffusion is the result of a significantly increased effective particle size 2*r*_heff_ = 30 nm for the PEO sample studied. This bound layer is not present in *sulfate* latex particles, which closely follow the Einstein-Stokes law for diffusion. The interaction between the *carboxylate-modified* particle surface and the PEO macromolecules has been confirmed by means of atomistic computer simulations. The grafted acrylate copolymers with a high surface/charge ratio confer more hydrophobic surface ready to interact with the polymer. The simulations suggest that the interaction is modulated by the nature of the acrylic acid monomer and the length of the grafted copolymer. More importantly, the atomistic simulation models developed in the present research allowed a clear interpretation of our experimental observations in terms of interactions between polymeric chains and nanoparticles. The results obtained point to the importance of computer simulations in determining the type of interactions that can take place between polymeric systems and particles. Thus, the combination of both the design of the nanoparticle together with the computer simulations may help in the selection of suitable nanoparticles for microrheological measurements. A couple of suggestions can be made regarding nanoparticle design, including the use of different types of materials (metallic, ceramic, polymeric, and hybrid nanoparticles), and different approaches for surface functionalization, as grafting with short copolymer chains and/or incorporating monomers with charged groups to dismiss the interactions between polymeric chains and nanoparticle surface.

## Supporting Information Available

Experimental details, polymer, and nanoparticles characterization (molecular weight, hydrodynamic size and electrophoretic mobility), and details about the construction of nanoparticle surface for simulations (PDF)
