# Supplementary material for: Revisiting Polymer–Particle Interaction in PEO Solutions
Source: Langmuir. 2021 Mar 25;37(13):3808–16. doi: 10.1021/acs.langmuir.0c02715 (PMC9132384; doi:10.1021/acs.langmuir.0c02715)

SUPPORTING INFORMATION

Revisiting polymer-particle interaction in PEO solutions

A. Espasa-Valdepeñas[1], J. F. Vega[1], V. Cruz[1] , J. Ramos[1] , A. J. Müller[2,3] and J. Martinez-Salazar[1]*

[1] Biophym, Departamento de Física Macromolecular, Instituto de Estructura de la Materia (IEM-CSIC) c/Serrano 113 bis, 28006 Madrid, Spain

[2] POLYMAT and Department of Polymers and Advanced Materials: Physics, Chemistry and Technology, Faculty of Chemistry, University of the Basque Country UPV/EHU, Paseo Manuel de Lardizabal 3, 20018 Donostia-San Sebastián, Spain

[3]IKERBASQUE, Basque Foundation for Science, Bilbao, Spain

1. Experimental details

1.1. Static light scattering

1.2. Dynamic light scattering

1.3. Electrophoretic mobility

2. Materials characterization

Figure S1. Debye plot of PEO sample from SLS measurements at T = 298 K. The error bars indicate the standard deviation obtained from at least 15 scans. The inlet shows the concentration dependence of the static intensity.

Figure S2. Squared electric field time autocorrelation function, $[g_1(t)]^2$, of the PEO sample solutions ($c \ll 1.5\ mg \cdot mL^{-1}$) at T = 298 K versus time at different concentrations. The inlet shows the concentration dependence of the hydrodynamic radius obtained from the cumulant analysis and the linear extrapolation to zero concentration.

Figure S3. (A) Squared electric field time autocorrelation function, $[g_1(t)]^2$, of the PS-SO_4^- particles (dotted lines, green and dashed lines, red) and the PS-CO_2^- particles (solid line, black) at T = 298 K versus time. **(B)** Intensity based hydrodynamic sizes of the systems.

Figure S4. (A) Phase plot and (B) electrophoretic mobility distribution of PEO sample (dash-dotted blue line) and PS particles (as in Figure S3) at T = 298 K.

3. Sulphate and carboxylated-modified polystyrene nanoparticles

Scheme S.1. Molecules used to build the bilayer systems

1. Experimental details

1.1. Static light scattering

The molecular weight and second virial coefficient of the PEO sample were determined by static light scattering (SLS). SLS data were obtained using the Zetasizer Nano ZS apparatus. The measurements for different sample concentrations in the dilute regime for solutions were obtained at T = 298 K. The results were fitted by linear regression to the Zimm equation [Zimm, B. H., The scattering of light and the radial distribution function of high polymer solutions. *J. Chem. Phys.* **1948,** *16*, 1093-1099]:

$$\frac{Kc}{R_\theta} = \left[\frac{1}{M_w} + 2B_{22}c\right]\frac{1}{P(\theta)} \quad (S1)$$

where M_w is the weight average molecular weight of the solute, B_{22} the second virial coefficient, and c its concentration in g·mL^{-1}. K is the optical constant given by:

$$K = \frac{4\pi^2 n_0^2}{N_a\lambda_0^4}\left(\frac{dn}{dc}\right)^2 \quad (S2)$$

where N_a is the Avogadro number, and dn/dc is the specific refractive index increment of the solution, which was set at 0.135 mg·mL^{-1}, the corresponding value for PEO, and the laser wavelength, λ_0 = 633 nm. The Rayleigh ratio, R_θ, was calculated by subtracting the solvent intensity from the solution intensity (I_A=$I_{Solution}$-$I_{Solvent}$) using toluene as the standard (I_T) for which R_T = 1.41×10^{-5} cm^{-1} at T = 298 K and λ_0 = 633 nm [Itakura, M.; Shimada, K.; Matsuyama, S.; Saito, T.; Kinugasa, S., A convenient method to determine the Rayleigh ratio with uniform polystyrene oligomers. *J. Appl. Polym. Sci.* **2006**, *99*, 1953-1959]:

$$R_\theta = \frac{I_A n_0^2}{I_T n_T^2} R_T \quad (S3)$$

where n_0 and n_T are the refractive index of the solvent and toluene, respectively. Each final R_θ data point was based on averaging not less than 10 statistically consistent measurements. Finally, P(θ) in Eq. (S1) is the shape factor, which embodies the angular dependence of the sample scattering intensity that occurs when the particles are big enough to accommodate multiple photon scattering. When the particles in solution are smaller than the wavelength of the incident light, multiple photon scattering is avoided. Under these conditions, the angular dependence of the scattering intensity is lost and P(θ) takes the value of 1 (in our case, as a first approximation, for spherical shells of expected radius of gyration, r_g, of around 20 nm, it takes a value of 0.90). The use of this simple approximation assumes that the third and higher virial terms contribute only negligibly to the measurements; this assumption can be considered valid

under the low protein concentrations used in our experiments. In addition to the SLS measurements, dynamic light scattering experiments have been performed at each concentration, in order to estimate the size of the polymer coils.

1.2. Dynamic light scattering

Dynamic light scattering (DLS) electric field correlations have been obtained for the polymer, particles and polymer-particles systems using the Zetasizer Nano ZS (Malvern Instruments) at T = 298 K. The Nano ZS instrument incorporates non-invasive backscattering (NIBS) optics and homodyne detection, to avoid artifacts in the determination of the size of the particles. This technique measures the time-dependent fluctuations in the intensity of scattered light that occur because particles in solution undergo Brownian motion. The intensity fluctuations measured are used to produce the scattered intensity time correlation function, $g_2(\tau)=\langle I(t)I(t+\tau)\rangle/\langle I(t)\rangle^2$, where I is the scattered light intensity and τ the lag time. The time-dependence autocorrelation function of the photocurrent was acquired in the solutions at variable concentration (c < 1.5 $mg\cdot mL^{-1}$) every 10 s, with 15 acquisitions for each run. The sample solution was illuminated by a λ_0 = 633 nm laser at a constant power, and the intensity of light scattered at an angle of θ = 173° was measured by an avalanche photodiode. The Siegert equation expresses the relation between the normalized time correlation function of the scattered intensity $g_2(\tau)$ and the normalized time correlation function of the electric field $g_1(\tau)$ [Berne, B. J.; Pecora, R., Dynamic light scattering with applications to chemistry, biology and physics. Dover Publications Inc.: Mineola, New York, **2000**]:

$$g_2(\tau) = B + \beta[g_1(\tau)]^2 \qquad \text{(S4)}$$

where τ is the lag time, B is the baseline and β (≤1) is a coherence factor that accounts for deviations from the ideal correlation and the experimental geometry. The corresponding cumulant for the field autocorrelation function can be written as:

$$g_1(\tau) = e^{(-\bar{\Gamma}\tau)}\left[1 + \frac{\mu_2}{2}\tau^2\right] \qquad \text{(S5)}$$

where $\Gamma = D_z \cdot q^2$ is the average decay rate with D_z being the z-average diffusion coefficient and $q = (4\pi n_0/\lambda_0)\sin(\theta/2)$ corresponding to the magnitude of the scattering vector, n_0 the refractive index of the solvent, λ_0 the wavelength of the laser, and θ the scattering angle. In Eq. (S6), μ_2 is the variance of the distribution, and the polydispersity index is defined as $Q = \mu_2 \cdot \Gamma^{-2}$. Once the decay rate Γ is obtained, it is used to obtain D_z and the z-average size by taking advantage

of the Stokes-Einstein relationship that relates Γ and, alternatively, the diffusion coefficient to the hydrodynamic radius, r_h, of the object that undergoes translational motion as:

$$r_h = \frac{k_B T}{6\pi\eta D} \qquad \text{(S6)}$$

In Eq. (S6), k_B is the Boltzmann's constant, T is the absolute temperature (T = 298 K), and η is the solvent viscosity, η = 0.889 cP [Kestin, J.; Sokolov, M.; Wakeham, W.A. Viscosity of liquid water in the range −8 °C to 150 °C. *J. Phys. Chem. Ref. Data* **1978**, *7*, 941-948]. This equation considers the particles as rigid spheres with a diameter related to the translational diffusion coefficient, which, in this instance, depends on the size and conformation of the particle at a given temperature and solvent viscosity.

1.3. Electrophoretic mobility

Electrophoretic mobility (EM) was measured in the Zetasizer Nano ZS apparatus, which uses phase analysis light scattering (PALS). In this application of the technique, a voltage is applied across a pair of electrodes placed at both ends of a universal disposable capillary cell containing the particle dispersion. The universal dip cell with palladium electrodes (Malvern Instruments ZEN1002) were used to perform the measurements. Charged particles are attracted to the oppositely charged electrode, and their velocity was measured and expressed per unit field strength as the EM, μ_e. The measurements were performed at T = 298 K. This type of analysis does produce EM distributions. Additionally, reduced voltages have been used (~ 5 V).

2. Materials characterization

The SLS results obtained for the PEO sample studied in this work are observed in **Figure S1**. The weight average molecular weight, M_w, of the sample has been estimated as 284.1 ± 8.0 $kg\cdot mol^{-1}$ and the second virial coefficient, B_{22}, as 7.5×10^{-4} $mL\cdot mol\cdot g^{-2}$. This later value is located in between those measured for monodisperse samples of similar M_w, $B_{22} = 1.6\times10^{-3}$ $mL\cdot mol\cdot g^{-2}$ [Devanand, K.; Sesler, J.C. Asymptotic behavior and long-range interactions in aqueous solutions of poly(ethylene oxide). *Macromolecules* **1991**, *24*, 5943-5947] and $B_{22} = 3.4\times10^{-4}$ $mL\cdot mol\cdot g^{-2}$ [Kawaguchi, S. Imai, G.; Suzuki, J.; Miyahara, A.; Kitano, T.; Ito, K. Aqueous solution properties of oligo- and poly(ethylene oxide) by static light scattering and intrinsic viscosity. *Polymer* **1997**, *38*, 2885-2891].

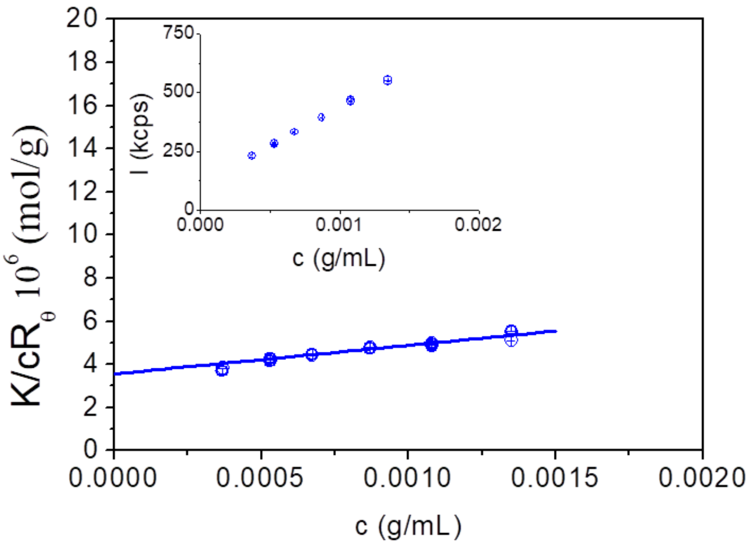

Figure S1. Debye plot of PEO sample from SLS measurements at T = 298 K. The error bars indicate the standard deviation obtained from at least 15 scans. The inlet shows the concentration dependence of the static intensity.

DLS results obtained for PEO sample within the concentration range explored ($c < 1.5$ mg·mL^{-1}) are suitable for the cumulant analysis given by Eq. (S5). The average diffusion coefficient obtained from this analysis is related to the so-called z-average hydrodynamic radius, r_z, upon application of Eq. (S6), which is an intensity-averaged size. The cumulant analysis gives value of the z-average sizes of $r_{z,0} = 28.6 \pm 1.3$ nm and mild polydispersity index of $Q = 0.37 \pm 0.03$ for PEO sample.

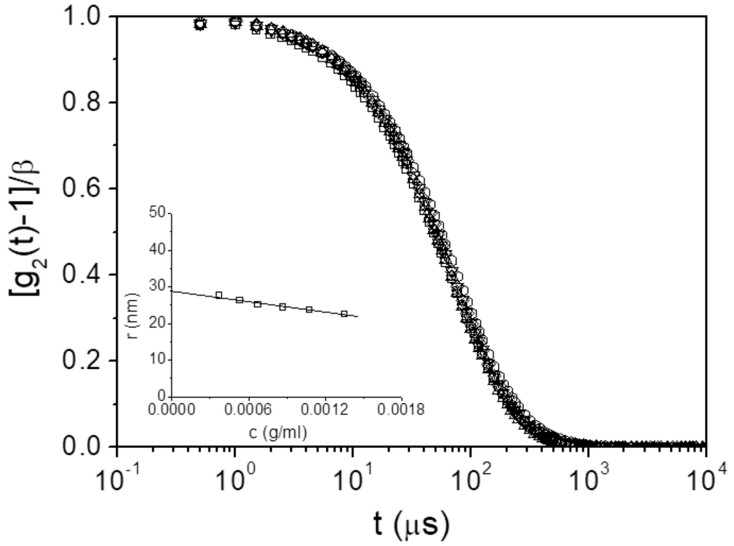

Figure S2. Squared electric field time autocorrelation function, $[g_1(t)]^2$, of the PEO sample solutions ($c \ll$ 1.5 mg·mL^{-1}) at T = 298 K versus time at different concentrations. The inlet

shows the concentration dependence of the hydrodynamic radius obtained from the cumulant analysis and the linear extrapolation to zero concentration.

DLS experiments have been also performed in the same conditions for the probe carboxylate ($PS-CO_2^-$) and suphate ($PS-SO_4^-$) particles. The expected results for monodisperse systems have been obtained, as it can be observed in **Figure S3**.

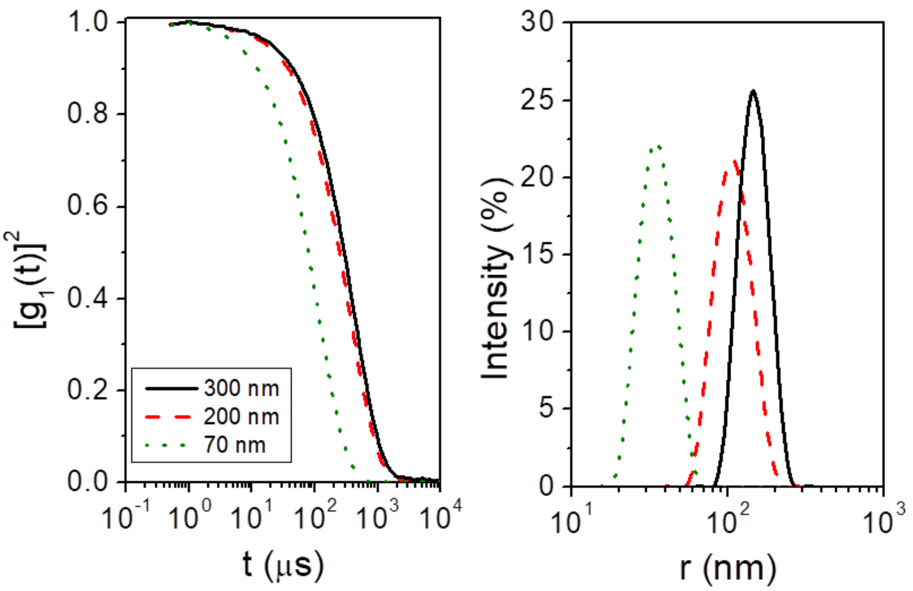

Figure S3. (A) Squared electric field time autocorrelation function, $[g_1(t)]^2$, of the $PS-SO_4^-$ particles (dotted lines, green and dashed lines, red) and the $PS-CO_2^-$ particles (solid line, black) at T = 298 K versus time. **(B)** Intensity based hydrodynamic sizes of the systems.

We have also obtained the values of the electrophoretic mobility, μ_e, of the PEO sample and the probe particles in water at T = 298 K. In **Figure S4** the results obtained in EM experiments (phase plot and μ_e) are given for the polymer and probe particles. The phase data show a nearly flat signal in the PEO sample and a low absolute value of μ_e, close to zero. For probe particles an increase of the amplitude in the oscillation of the signal is clearly seen. Since the PEO polymer chains have no chemically bound charges, the small negative charge detected in the measurements is likely caused by few ions physisorbed to the PEO chains. The particles show characteristic high absolute values of μ_e, as expected for stabilized colloidal systems, which is due to the presence of dissociated functional charged groups at the surface.

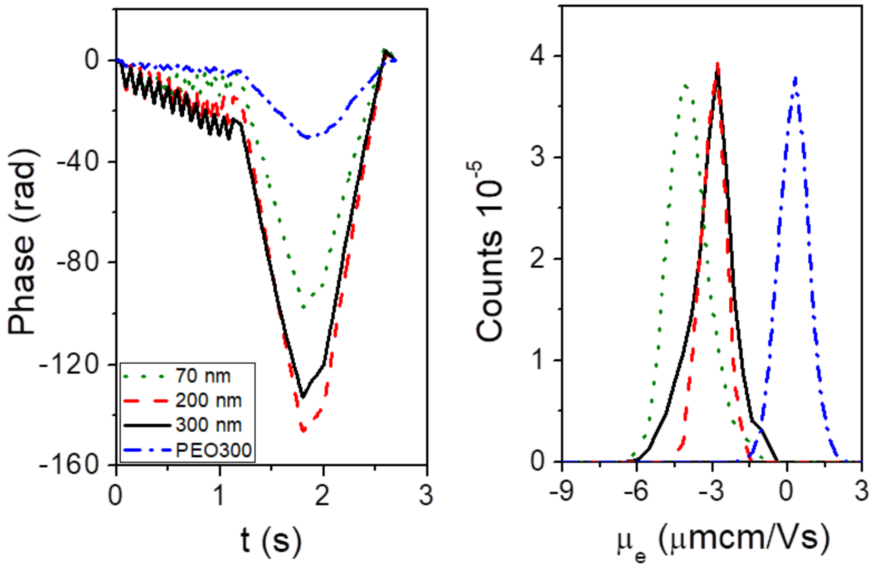

Figure S4. (A) Phase plot and (B) electrophoretic mobility distribution of PEO sample (dash-dotted blue line) and PS particles (as in Figure S3) at T = 298 K.

3. Sulphate and carboxylated-modified polystyrene nanoparticles

Polystyrene (PS) based nanoparticles are usually prepared by emulsion polymerization. Zhao and Brown briefly described the technique [Zhao, J. X.; Brown, W., Surface Characteristics of Polystyrene Latex-Particles and Comparison with Styrene-Butadiene Copolymer Latex-Particles Using Dynamic Light-Scattering and Electrophoretic Light-Scattering Measurements, J. Colloid Interf. Sci. **1996**, 179 (1), 255-260]. This procedure suggests that SDS molecules in the micelle can remain trapped on the nanoparticle surface with the sulfate moieties exposed to the hydrophilic phase. Atomistic molecular dynamics simulations show the insertion of the SDS molecule on the interfacial surface so that the hydrophobic hydrocarbon chain remains in the polymer hydrophobic core whereas the sulfate group locates in the polar solvent medium [Li, Z.; Van Dyk, A.K.; Fitzwater, S.J.; Fichthorn, K.A.; Milner, S.T. Atomistic Molecular Dynamics Simulations of Charged Latex Particle Surfaces in Aqueous Solution. Langmuir **2016**, 32 (2), 428-441; Li, Z.; Fichthorn, K. A.; Milner, S. T. Surfactant Binding to Polymer-Water Interfaces in Atomistic Simulations. Langmuir **2016**, 32 (30), 7519-7529]. Coarse grained simulations also point to the hydrophobic polymer particle surface saturation with surfactant molecules [Wang, S.; Larson, R.G. Coarse-Grained Molecular Dynamics Simulation of Self-Assembly and Surface Adsorption of Ionic Surfactants Using an Implicit Water Model. Langmuir **2015**, 31 (4), 1262-1271]. It can be expected, therefore, a close pack of sulfate anions on the surface of the nanoparticle synthesized by the procedure described above.

In the nanoparticles produced by the common procedure described above a small PA should be expected from the fact that many SDS molecules would be trapped on the particle surface along

with the sulfate groups at the ends of the polymer chains coming from the polymerization process. The carboxyl functionalized nanoparticles are built by grafting a copolymer composed by different aliphatic acrylates, usually butyl acrylate (BA), methyl methacrylate (MMA) and methacrylic acid (MAA) on the PS nanoparticle. The acrylic acid groups form a kind of multianionic "hairs" spread over the nanoparticle surface. The number of charges increases respect to the non-functionalized nanoparticle due to the contribution of each charged monomer. In principle, the resulting PA will be lower due to the increased charge number. However, at the same time, the accessible surface increases too due to the exposition of the copolymer chains to the hydrophilic phase. It has been already shown by atomistic scale simulations that PEO macromolecules are able to shield hydrophobic surfaces from polar solvents as is the case for certain peptides [Jones, M.N. The interaction of sodium dodecyl sulfate with polyethylene oxide. J. Colloid Interf. Sci. **1967**, *23* (1), 36-42]. PEO also adsorbs on the SDS micelle surface forming "bead-necklace" structures described as a hydrophobic interaction between the polymer and the accessible SDS hydrocarbon tails of the micelle [Shang, B.Z.; Wang, Z.; Larson, R.G. Molecular Dynamics Simulation of Interactions between a Sodium Dodecyl Sulfate Micelle and a Poly(Ethylene Oxide) Polymer. J. Phys. Chem. B **2008**, 112 (10), 2888-2900].

Scheme S.1. Molecules used to build the bilayer systems

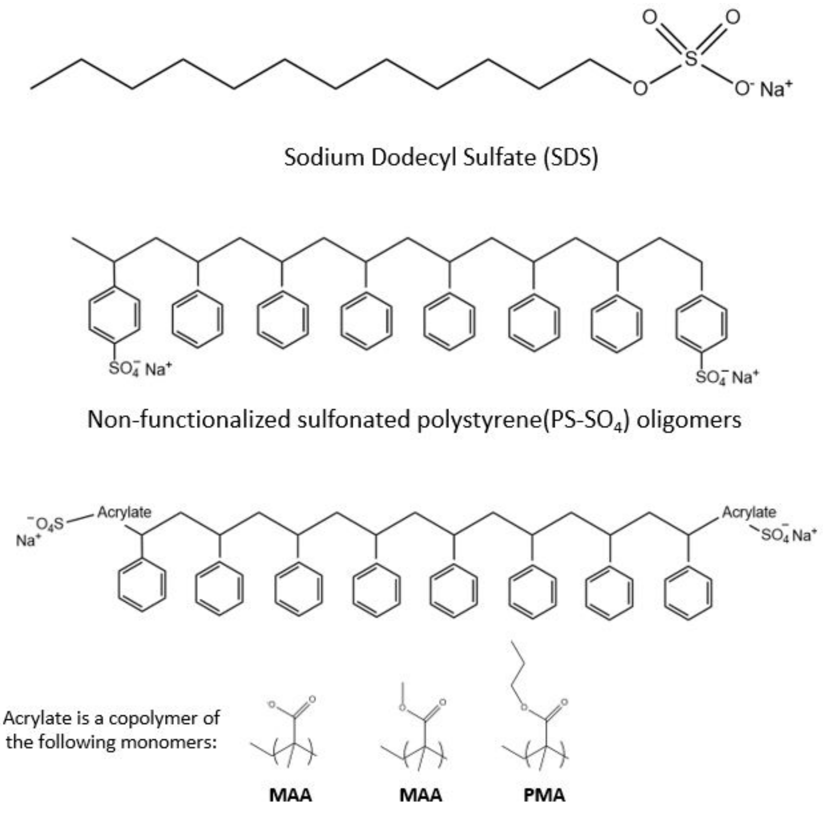

Supplement: Supplementary file 1 — la0c02715_si_001.pdf [file la0c02715_si_001.pdf]
